# The future in the litter bin – bioconversion of food waste as driver of a circular bioeconomy

**DOI:** 10.3389/fnut.2024.1325190

**Published:** 2024-05-06

**Authors:** Afraa Razouk, Eduard Tiganescu, Anthea Julia von Glahn, Ahmad Yaman Abdin, Muhammad Jawad Nasim, Claus Jacob

**Affiliations:** Division of Bioorganic Chemistry, School of Pharmacy, Saarland University, Saarbruecken, Germany

**Keywords:** food biomass, anaerobic digestion, composting of food waste, insect-based-conversion, circular economy, sustainability, biofuels

## Abstract

Bioconversion of organic waste requires the development and application of rather simple, yet robust technologies capable of transferring biomass into energy and sustainable materials for the future. Food waste plays a significant role in this process as its valorisation reduces waste and at the same time avoids additional exploitation of primary resources. Nonetheless, to literally become “litterate”. extensive research into such robust large-scale methods is required. Here, we highlight some promising avenues and materials which fulfill these “waste to value” requirements, from various types of food waste as sustainable sources for biogas, bioethanol and biodiesel to fertilizers and antioxidants from grape pomace, from old-fashioned fermentation to the magic of anaerobic digestion.

## 1 Introduction

In retrospect, future generations may look back at the first half of the 21^st^ Century as a watershed, not only in politics, but also in economy and especially in the way we allocate and manage our limited resources and generate energy (1). If the present years turn out to be a truly epochal change, as some have already claimed, or just as another small milestone in a slow yet continuous transformation toward a carbon neutral and fairer world, needs to be seen (2). In any case, change is on the horizon and there is a rising demand – and interest – in new technologies and their various practical implementations (2, 3).

Our economic activities and practices during the last decades have indeed placed tremendous pressure on the ecological boundaries of planet Earth and are now threatening to ravage nature’s delicate balances and eco-systems. The constant drive toward growth and economic efficiency at the cost of society and the environment must be reconsidered (4, 5). As of 2022, the global estimate of atmospheric CO_2_ is around 414 ppm which exceeds the proposed planetary limit by over 60 ppm already. Fertilizers rich in nitrogen and phosphorous are being employed with amounts double what the soil, surface water and various species can tolerate (4, 5). It is expected that more than 100 species are being extinct every day and one should embrace the fact that we ourselves are also just such a species and we certainly can be healthier and living better within a healthy environment (4, 5).

When considering sustainability of our limited natural resources, several strategies come to mind, such as the 5Rs, i.e., refuse, reuse, refine, recycle or revaluate. We believe this list can be extended further to even 10 such Rs, including remove, reduce, repair, replace and rally (6–9). It is, for instance, possible to reduce dependence of or to even entirely refuse a plastic bag at the supermarket or to reuse it a couple of times for different purposes (10, 11). The plastic bag can be even removed and replaced with a more sustainable alternative such as a cotton bag which can be repaired and rallied for as an item worth revaluation. It is also possible to refine petrol engines so they consume less gasoline (12, 13). Some plastics may even be recycled whereas others, such as yogurt beakers, can be decorated with a lick of paint and thus re-valued as decorative flowerpots (14).

Yogurt beakers besides, however, one major source of potential value comes from waste itself, especially in form of wasted food and food waste. This material is of particular interest, since it is organic, renewable and available in large quantities at low or no costs.

Indeed, food waste may originate at any point during the complex modern food production system stretching from the “farm to the fork” (15). Whether at production, processing, distribution, retail or consumption, unused leftover materials are not only considered loss and waste, they also pose a serious ecological hazard. The composition of food waste varies depending on the source and can be categorized as pre-consumer and post-consumer food wastes (16). The pre-consumer food waste usually contains higher amounts of fruit and vegetable (48%), root and tubers (26%), cereals (14%) and lower amounts of dairy (6%), meat (3%), oil (2%) and fish (1%) based on dry weight. The post-consumer food waste primarily comprises of cereals (52%), fruits and vegetables (21%), dairy (12%), lower content of meat (6%), roots and tubers (6%), oil (2%) and fish (1%) of the dry weight (16). Similarly, the content of nutritional substances such as carbohydrate, fat, protein, starch, hemicellulose and non-nutritional products such as lignin also varies not only for pre- and post-consumer food wastes but also for different types of wastes within these two categories (16). Pre-consumer apple waste, for instance, contains carbohydrates (48.1%), hemicellulose (24.4%), lignin (23.5%) and cellulose (7.2%) and the pre-consumer grape waste contains hemicellulose (30.3%), starch (21.0%), lignin (17.4%) and proteins (6.1%) (16, 17). The post-consumer food waste varies for its nutritional composition. Kitchen garbage comprises of fats (18.0%), carbohydrate (16.0%) and proteins (15.6%) (16, 18). Moreover, post-consumer used frying oil comprises of carbohydrates (28.4%), proteins (21.6%), fats (19.4%) and cellulose (3.9%) (16, 19). Post-consumer food industry waste such as grape pomace primarily comprises of proteins (43–75%) and lipids (6–15%) (16, 20).

According to the Food and Agriculture Organization (FAO), more than 17% of the total food produced globally becomes waste each year. In 2022, 2% were lost in retail, 5% from food services, and 11% were wasted by households (21). It is important to mention that the size of population is a crucial determinant which influences the volume of food waste generated within a country. Nations with larger populations tend to produce higher amounts of food waste (22, 23). In 2023, India and China, represent the world’s most populous countries with approximately 1,428,627,663 and 1,425,671,352 inhabitants, respectively. China’s annual food waste amounted to a staggering 91.65 million metric tons, while India generated approximately 68.76 million metric tons for 2020 (22, 23). Conversely, Germany, with a population of 83,294,633, produced a comparatively lesser amount of, approximately 6.26 million metric tons of food waste in 2020 (22, 23).

In Germany, household food waste alone currently amounts to a staggering 75 kg per adult per year, often to the delight of *Rattus norvegicus*, the brown rat (24). Indeed, food waste undermines the sustainability of our food systems as all the resources invested into producing this food, such as land, energy, and water, are in one way or another “wasted” (25, 26). Furthermore, the disposal of food waste in landfills or through incineration, besides attracting vermin, also contributes to greenhouse gas emissions by 8%, thereby exacerbating climate change (25, 27).

In contrast, bioconversion can be used to give such waste a renewed purpose, for instance to produce biofuels, fertilizers, animal feed, and nutraceuticals (25, 27). This approach offers a sustainable means to utilize food waste and reduce its environmental impact (28). It, therefore, hardly comes as a surprise that a future circular economy probably has to be based to a large extent on renewable materials and energies, among which organic natural materials are likely to play a dominant role (29–31). Apart from especially cultivating plants for energy and materials, which requires land, energy, effort and labor, organic waste - a cornucopia of resources if treated properly and with respect - only needs to be collected (32). Not surprisingly, our attitudes about traditional waste are currently undergoing major changes. To put it simple: *Waste is simply a valuable resource at the wrong place, at the wrong time and in the wrong hands* (33–35). It is time to close the circle with the missing stretch from “the fork to farm”.

With this new attitude in hand, novel research topics and strategies have come into view in order to unlock the potential of such biological waste-resources as part of a future circular bioeconomy (33, 35). In this review, we shall showcase a few rather stimulating projects aiming to unlock the potential of organic food waste for new sources of (old) materials. The overarching attraction of these strategies results from a combination of (a) harvesting raw materials, (b) efficient waste management and (c) local economic circles. Rather than presenting a comprehensive review, though, we shall focus on a few selected and indeed elegant examples which we consider equally instructive and stimulating (36).

## 2 Aerobic decomposition (composting, vermicomposting and insect based-bio conversion)

As the litter rat has already been mentioned, the compost heap is the first port of call when it comes to processing food waste. Aerobic decomposition is an eco-friendly process to convert organic waste into fertilizers for soil. This method requires specific conditions such as an amenable carbon to nitrogen ratio (the ideal C to N ratio ranges from 25:1 to 40:1), moisture content (40–60%), aeration, pH, the mix of feedstock materials and, of course, microorganisms (37).

Composting can be divided into three main phases. The first phase, known as the mesophilic phase, is carried out by mesophilic microorganisms such as bacteria (*Pseudomonas* and *actinobacteria*) and fungi (*Penicillium* and *Aspergillus*) (38–40). In this phase, the microorganisms break down the organic compounds such as sugars and starch into simpler molecules with production of carbon dioxide (CO_2_) and water. The mesophilic phase lasts for 2 days. The second phase is the thermophilic phase, which occurs as the temperature rises to reach 55°C to 77°C due to bacterial and fungal activity. Decomposition is now facilitated by thermophilic microorganisms such as *Thermus aquaticus* and *Streptomyces coelicolor* (41). Here the thermophilic bacteria break down the more complex organic compounds such as fats, cellulose and proteins to produce carbon dioxide (CO_2_) and water. This phase lasts for a few weeks to several months. Third, follows the maturation phase, which is characterized by a drop in the temperature, with mesophilic bacteria such as *Actinobacteria* and fungi such as *Mucor* and *Trichoderma* dominating the process (38–40). In this phase, after the waste pile cools down and mesophilic microorganisms become active again, the remaining organic matter continues to break down until the pile stabilizes. Then, the compost matures and over time becomes similar to the normal traditional soil. This phase lasts for several months (42). Subsequently, the resulting compost requires a pathogen removal process such as exposure to direct sunlight for drying, steam treatment and pasteurization (at around 60°C to 71°C) (43). The pathogen removal step is important to minimize the risk of spreading various plant and animal diseases, such as histoplasmosis, aspergillosis, tetanus, paronychia, and hypersensitivity pneumonitis (Farmer’s lung). On the other hand, several studies have affirmed a natural antimicrobial inhibitory effect of compost products, e.g., tea compost, against certain phyto-pathogens, such as *Pythium debaryanum*, *Rhizoctonia* species, and *Fusarium oxysporum* (40). In any case, it is actually counterproductive to try to sterilize food waste prior to composting, as has been suggested in the past, especially because of *Aspergillus*.

**Figure 1:**
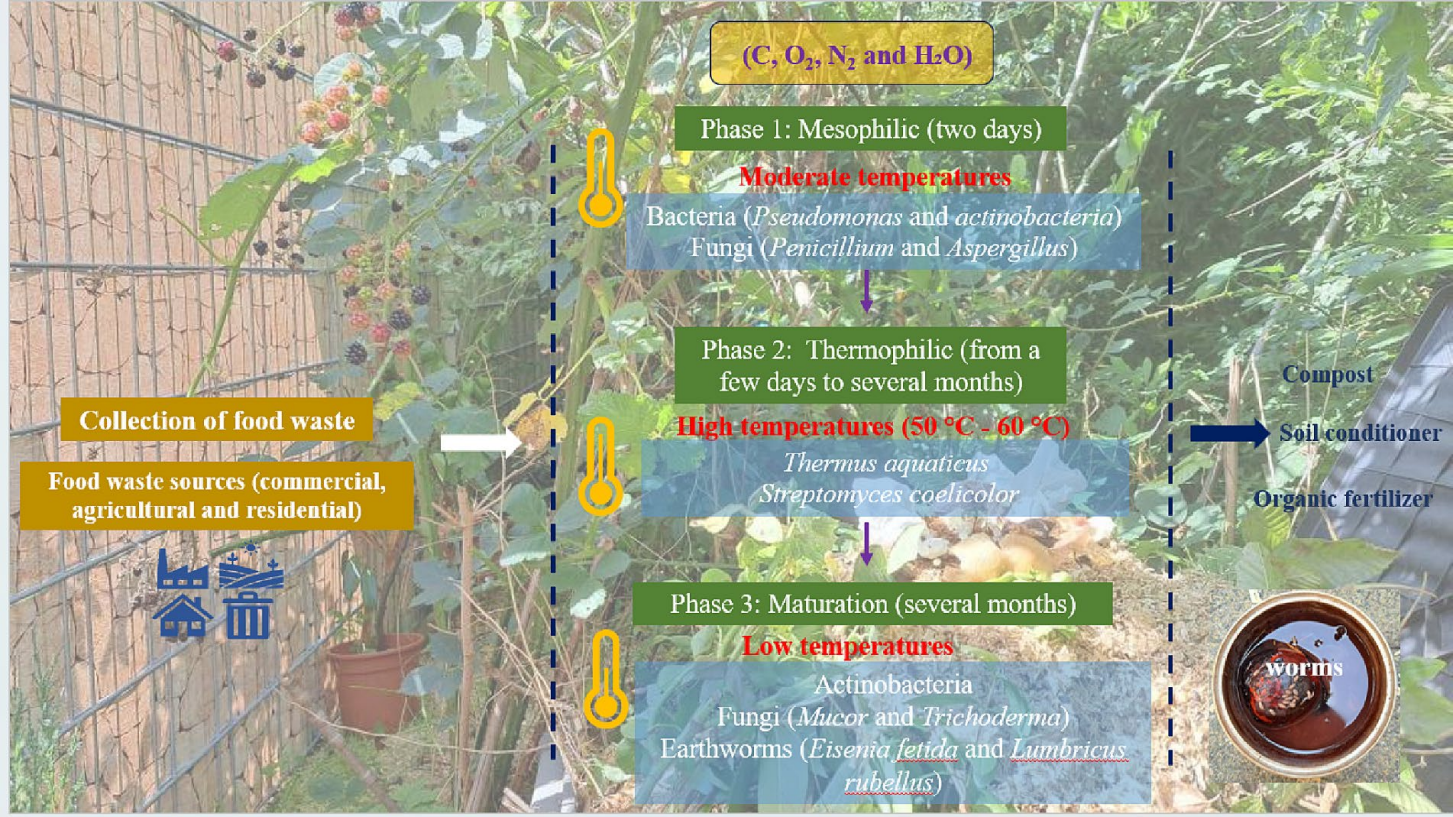
Phases of composting including the relevant microorganisms: mesophilic phase, thermophilic phase and the final maturation phase to produce soil compost, conditioner and fertilizers. Photographs were created by © 2024 Elizabeth Jacob.

Besides producing a valuable organic material which nowadays replaces peat in gardening and agriculture, composting additionally benefits the environment by reducing residential food waste and lowering greenhouse gas emissions. As for the “logistics”, composting can be achieved easily in a highly decentralized fashion, for instance at home, and thus does not require collection and transportation of materials, although collections and centralized facilities are also very common, especially in urban areas. Curiously, some German cities, such as Sankt Ingbert, even employ professional “compost inspectors” who will check your personal heap and either approve it or force you to take part in the city’s centralized “green bin” collection system (44–46). Indeed, different composting methods for organic waste are amenable, such as aerated static pile composting, in-vessel composting and mound bed composting (the famous Germanic “Hügelkultur”) (47–50). Figure 1 briefly illustrates the composting process (44–46).

Another type of composting is known as “vermicomposting” by using earth worms to convert food waste into soil fertilizer. In this composting method the commercial, agricultural and residential food waste are passed through the worm-gut to form a nutrient rich vermicompost (51). The vermicompost - or worm castings - can be easily implemented indoors, in small batches with less nitrogen loss, and even in the cold weather. It is the richest nutrient fertilizer known to gardeners (52). People who are interested in growing their own vegetables are becoming interested in earthworm castings (52, 53). After the vermicompost or worm castings feed on the food waste, the worms produce particles which improve the soil properties and increase the water retention within the soil. The vermicompost contains phosphorous (1.8–2.2%), potassium (1.0–1.5%), nitrogen (1.5–2.2%) and micronutrients such as iron, magnesium, sulfur, zinc, calcium and sodium. More details on vermi-worm-compost are presented in Table 1 (51, 62). It is important to mention that well-managed vermicomposting systems are also aerobic and no worms are being hurt, thus vermicomposting can create a sustainable environment for earthworms to thrive and continue their life cycle.

**Table 1:** Composting and vermicomposting.

Key differences	Composting	Vermicomposting	References
Organisms	Aerobic microorganisms such as bacteria and fungi	Earth worms such as *Red Wiggler* (*Eisenia foetida* or *Eisenia andrei*) and *Lumbricus rubellus*	(54)
Microbial activity	Breaking down of the complex organic compounds into simpler substances by microbes	Breaking down complex molecules by specialized enzymes in the gut of the worms	(55)
Temperature and speed	Higher temperatures	Lower temperatures	(56)
Odor	Higher odor	The presence of worms maintains suitable aeration and less unpleasant odor	(57)
Space required	More	Less	(58)
Plant growth regulator	Absent	Present	(58)
End products	Compost or humus	Vermicompost or worm castings	(51)
Nutrients in the end product (%) Calcium (Ca) Copper (Cu) Iron (Fe) Manganese (Mn) Magnesium (Mg) Nitrogen (N_2_) Organic carbon Phosphorus (P) Potassium (K) Sodium (Na) Zinc (Zn)	2.27 0.0017 1.1690 0.0414 0.57 0.8 9.8–13.40 0.350.48 0.01 0.0012	1.18–7.61 0.0026–0.0048 0.2050–1.3313 0.0105–0.2038 0.093–0.568 0.51–1.61 12.2 0.19–1.02 0.15–0.73 0.058–0.158 0.0042–0.110	(51, 59)
Process costs	Low	High	(54, 60)
Advantages	Higher temperatures lead to pathogen free and rather clean final product Waste reduction Nutrient soil fertilizer and amendment	Faster than traditional composting Less space required Richer compost Less odors	(45, 54, 60, 61)
Disadvantages	Uncontrolled emissions of methane Release of odors Requires more space Water pollution	Worms are sensitive to temperature which may affect the whole process in case of extremely cold or hot climates More useful for low volumes of food waste due to limited capacity of vermicomposting bins	(45, 54, 60, 61)

Notably, vermicomposting differs from normal composting in several ways. The temperature in vermicomposting, for instance, ranges from 10°C to just 32°C and the process is faster than microbial composting because the food waste passes through the earthworm gut, where a significant but not fully understood transformation takes place, enriching the resulting earthworm castings (worm manure) with plant growth regulators (51, 54, 55). Furthermore, earthworms produce a richer kind of compost, and thus are capable of transforming the food garbage into “gardeners’ gold” (51, 63).

The quantity of CO_2_ released from composting and vermicomposting varies for different waste systems as well as the organisms involved. In terms of aerobic digestion, anaerobic digestion and vermicomposting, CO_2_ constitutes the largest portion of greenhouse gas emissions that is 63.6, 80.9 and 78.3%, respectively (64–66). The main differences between composting and vermicomposting are also summarized in Table 1.

Amazingly, besides bacteria, fungi and worms, even insects can be utilized to convert food waste into soil fertilizers (67). Larvae of more than 2,000 species of insects participate in such conversions and include, for instance, the larvae of black soldier flies (*Hermetia illucens*), two-spotted crickets (*Gryllus bimaculatus*), desert locusts (*Schistocerca gregaria*), house flies (*Musca domestica*), Cambodian field crickets (*Teleogryllus testaceus*), yellow mealworms (*Tenebrio molitor*) and others (see Figure 1, insert) (68–72). Insects are employed already in such processes for a number of advantages which range from being cost effective to the possibility of reducing waste odors compared to other methods (73–75).

The process of insect bioconversion begins with the collection of food waste from residential areas, farms, or restaurants. Subsequently, the collected waste is placed into certain reactors such as bins, cages, vertical farming systems, insect bags and sleeves, modular systems or insect trays (76). After that, it is exposed to species such as the black soldier fly (BSF) larvae, which can devour fruits, vegetables, and various organic waste into soil fertilizers. The larvae consume the organic waste as part of their natural feeding source, converting waste into a biomass after breaking down the organic materials within their digestive systems (75, 76). Once the insects have completed their life cycles and reached their desired stage of development the biomass can then be collected and used as nutrient-rich soil amendment and fertilizer (77). The biomass produced by such insects generally is rich in nutrients such as N, P, K, Ca, Mg, S, as well as microelements, like Mn, Na, B, Zn, Fe, and Cu (75). It is important to mention that after producing the fertilizers, the insects are not intentionally killed and they continue their life cycles. Different insect species can be used but BSF is the most common choice due to its ability to reduce organic waste biomass by 60%, transforming it into nutrient-rich biomass (69, 75). This method is environmentally friendly and sustainable as it helps reduce waste volume and greenhouse gas emissions. Also, BSF, two-spotted crickets or desert locust larvae have the capability to produce high-quality fertilizers known as frass fertilizers (70). These insect-driven processes could yield a variety of beneficial materials besides fertilizers, such as precursors of bioplastics (67, 74). Table 2 provides a more comprehensive list of insect species suitable for bioconversion of food waste.

**Table 2:** Bioconversion of food waste by variety of insect species.

Insect		Food wastes	Countries	Bioconversion approach	References
Scientific names	Common names				
*Hermetia illucens*	Black soldier Fly (BSF)	Rice straw, restaurant waste	China	Biofuel	(76, 78)
		Coffee pulp, husk	Indonesia	Biomass	(76, 79)
		Peels from fruit industry (banana and orange)	Sweden	Soil enhancement	(80)
*Gryllus bimaculatus*	Two-spotted cricket	Soybean, sweet potato and wheat bran	Kenya-Uganda	Frass fertilizer	(69, 73)
*Schistocerca gregaria*	Desert locust	Wheat bran, barley and wheat seedlings	Kenya- Uganda	Frass fertilizer	(72, 73)
*Musca domestica*	House fly (HF)	Restaurant waste and corn silage	China	Biomass, biofuel, fertilizer	(76, 81)
*Teleogryllus testaceus*	Cambodian field crickets	Cassava plant tops, spent grain, mung bean sprout waste	Cambodia	Biomass	(76, 82)
*Acheta domesticus*	House cricket	Food waste	Japan	Digestion of municipal food waste	(70)
*Tenebrio molitor*	Yellow mealworm	Corn stover	China	Biofuel	(76, 83)

In this context, one may also mention that in the past, food waste has been fed to many higher animals, from rabbits to pigs and cows, and that in some instances, these animals have become seriously ill. The transmission of “Mad Cow Disease”, for instance, has been due to cows being fed meat-based products. Thus employing higher animals rather than microbes, worms and larvae for such bioconversions may be a considerably more controversial issue.

## 3 Anaerobic digestion

Aerobic digestion is an energy intensive process which comsumes a lot of energy and air to produce CO_2_ instead of methane. As far as the energy balance is concerned, it is therefore often more economical to digest anaerobically. Here, organic waste from the food industry, such as fruits and vegetable waste, rice and maize straw and coffee husk, form the basis for bio-methanation thanks to their intrinsic methanation potential (84, 85). In fact, anaerobic digestion (AD) is one of the most common industrial-scale processes to convert food waste into renewable materials using “biological” means, in contrast, for instance, to pyrolysis. AD encompasses several phases: the process starts with preparing the feedstock where food waste is collected and sorted to remove the non-biodegradable materials. Then, the selected food waste is added into a large tank called “anaerobic digester” in the absence of dioxygen (86).

**Figure 2:**
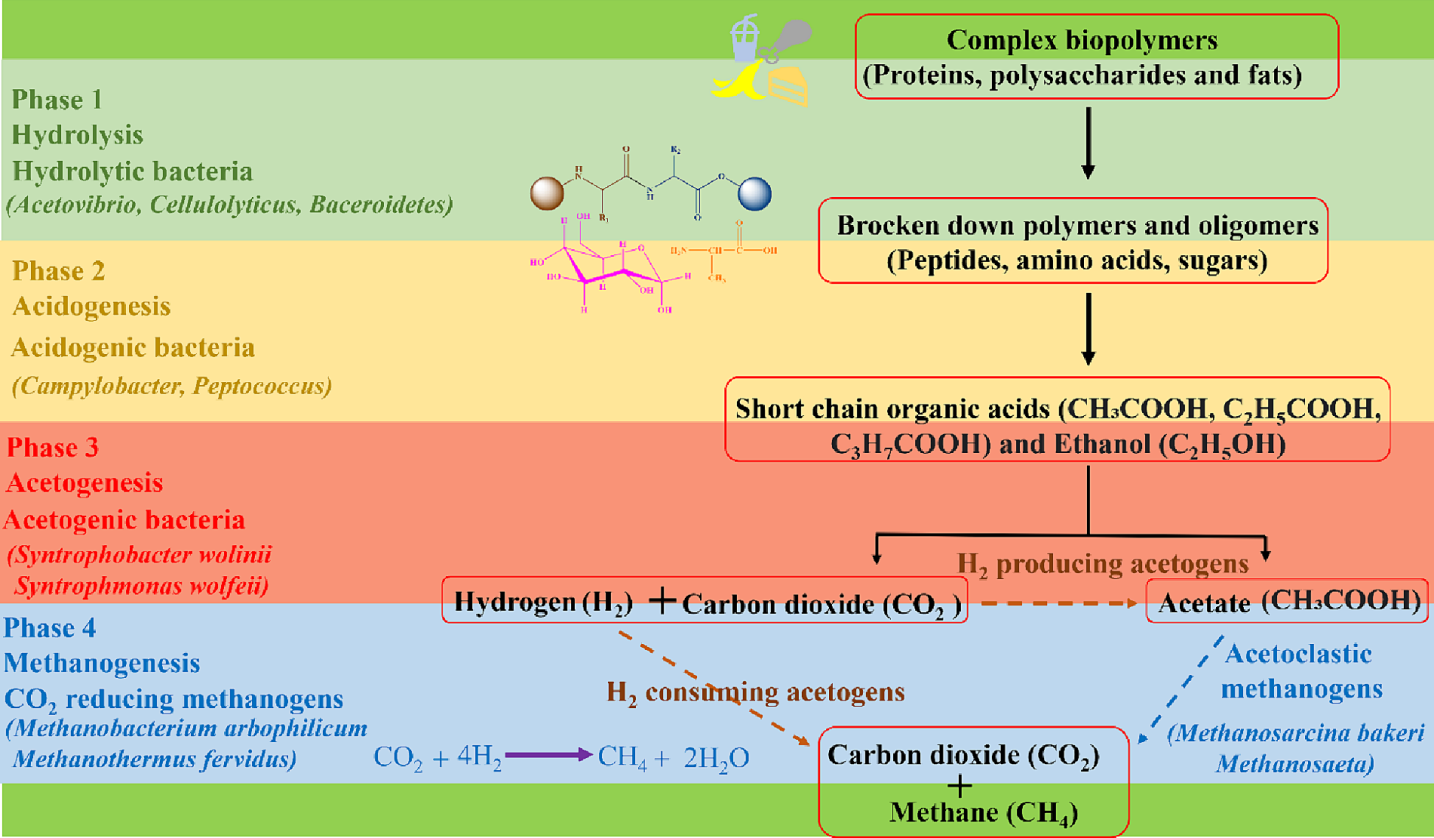
The complex biopolymers undergo Anaerobic Digestion (AD) which involves a series of biological processes in the presence of microorganisms to produce biogas.

Once inside the digester, the digestion process begins through microbial activity of specialized bacterial communities which break down food waste in successive, different phases. In the first phase, hydrolysis occurs, facilitated by hydrolytic bacteria such as *Bacteriodes*, *Clostridium*, and *Acetivibrio* (86, 87). These bacteria break down food waste into its basic building blocks, e.g., amino acids, sugars, alcohols, and fatty acids at a pH of around 6.5 to 7.5. In the second phase, called acidogenesis, acidogenic bacteria such as *Enterobacterium*, *Acetobacterium*, and *Eubacterium* transform the hydrolysed materials into intermediate products, such as acetate, propionate, ethanol, lactate, and volatile fatty acids at pH ranging from 5.5 to 6.5 (86, 88). In the third acetogenesis phase, acetogenic bacteria such as *Syntrophomonas wolinii* and *Syntrophomonas wolfeii* utilize acidogenic products to produce acetic acid (CH_3_COOH), carbon dioxide (CO_2_) and hydrogen (H_2_) at a pH ranging from 6.2 to 6.8 (86, 89). Eventually, acetoclastic methanogens such as *Methanosarcina barkeri*, *Methanosarcina frisius*, and *Methanobacterium formicicum* come into play and convert acetate to methane (CH_4_), CO_2_ and water (H_2_O) (86, 90). Additionally, hydrogenotrophic methanogens such as *Methanobacterium arbophilicum* and *Methanothermus fervidus* convert H_2_ and CO_2_ into predominantly CH_4_, thus contributing further to the production of CH_4_ and scavenging some of the CO_2_ at a pH ranging from 6.5 to 7.5 (86, 90). Biogas produced from AD comprises of 50–75% CH_4_ and 50–25% CO_2_ concentration. The flow of materials and different stages of AD are presented in Figure 2 (91).

The choice of the most suitable microorganisms is important for successful AD and lends itself to further research and development. Interesting examples of such anaerobic bacterial species employed in AD for biogas production include *Clostridium kluyveri, Methanosaeta harundinacea,* and *Propionibacterium propionicus*, among approximately a thousand other species (86, 92). Notably, certain bacteria, such as *Selenomonas ruminantium, Rhodopseudomonas palustris P4, Citrobacter* spp. *y*19, and *Clostridium butyricum*, are capable of producing “green” H_2_ in place of CH_4_, which can be utilized directly or converted chemically into biogas (93). Indeed, the topic of fermentative H_2_ production has gained significant attention recently not only in research but also in politics and legislation as part of the “net zero” strategy of switching the traditional carbon-based to a new hydrogen-fuelled economy (92, 94–96). As for numbers, Germany is one of the leading biogas producers globally with 28.8 million m^3^ of raw biogas generated and fed into the gas grid annually which is indeed a step into the right direction considering the huge demand and usage of more than 77 billion m^3^ in 2022. According to the International Energy Agency (IEA), China has established the highest number of biogas plants (more than 100,000 plants), followed by Germany (more than10,000 plants). In comparison, there were less than 350 Anaerobic Digesters operating in the United States in 2022 (97, 98). AD is, however, associated with a few disadvantages, such as the emission of significant amounts of CH_4_ and the release of odors during production. Moreover, AD necessitates a careful equilibrium between the energy consumed during the operating process and the energy gained (45, 86, 99, 100). The costs associated with the AD can vary significantly depending on several factors such as the feedstock type, the size of the AD system-whether it’s a small-scale on-farm digester or a large municipal facility- and the use of mesophilic or thermophilic digestion equipment and infrastructure required for the transportation of the biogas produced may contribute additional costs (86, 99, 101, 102). Furthermore, the cost of land and infrastructure for AD should also be considered (86, 101). The total capital cost for the AD in Tollenaar Holsteins Dairy, California, United States was US$1.7 million, with annual operating and maintenance costs of 50,000 USD (103). Butler Farms, Texas, USA implemented a lagoon AD with costs ranging from 550,000 USD to 650,000 USD with an annual maintenance cost of 25,000 USD (103). Fair Oaks Dairy Indiana, United States, employed a mixed-plug flow AD costing 12 million USD with estimated annual operating and maintenance costs of around 600,000 USD (103).

In sharp contrast to aerobic digestion, which focusses on the production of compost and thus ignores the effluvium, the products of AD can be divided into two main categories, biogas and digestate. Biogas can be utilized directly, for instance for heating, as vehicle fuel or to generate electricity. Digestate, on the other hand, is still rich in nutrients and can be converted further to biofertilizers for agricultural use (104). Compared to composting, AD entails higher costs, both in construction and operation, yet has the benefit of producing clean energy and heat, providing increased value to EU countries that may import most or all of their fossil fuels (105, 106). Recent advancement in the field of AD includes, anaerobic co-digestion (AcoD), where different organic waste products can be combined and blended with various other substrates. Additionally, soft magnetic ferrite serves as an effective and economical AD additive which not only promotes biomethane production but also recovers the materials and microorganisms (107, 108). Moreover, decentralized AD which operates in smaller-scale facilities gets rid of food waste generated from restaurants, cafeterias, cooked food centers or households (109).

### 3.1 Digestate

After the completion of the AD process, the remaining materials from each step are collected together as a whole digestate and exploited as a fertilizer (110, 111). Digestates can originate from different sources, such as maize, clover, corn, coffee, tea, fruits, stems, bakery waste, and sugar industrial waste. They are generally rich in potassium, ammonium and phosphorus (111). Phosphorus is found in food waste such as grains, dairy, and meat. Similarly, nitrogen is also generated during the AD process from proteins and other organic compounds, and it is converted into ammonia (NH_3_) (112, 113). A relevant example of a digestate rich in phosphorous and nitrogen is the one generated from algal biomass. Such digestate contains around 390 mg/L of total phosphorous and 2,940 mg/L ammonia-nitrogen among other elements such as sodium and potassium (113, 114).

Such digestate is used primarily as a soil fertilizer and in hydroponic systems in greenhouses (111). It has been reported that applying the digestate in bubble-insulated greenhouses leads to higher yields of vegetables, such as lettuce, cape gooseberries, tomatoes, cucumbers, mushrooms (such as *Agaricus subrufescens* and *Agaricus arvensis*), and herbs (such as thyme, dill, parsley, basil, coriander, and melissa) (115). Moreover, the utilization of AD digestate as a fertilizer has demonstrated its ability to induce resistance against plant diseases and soil-borne pathogens, such as *Fusarium oxysporum f.* spp. *Spinaciae, Ralstonia* spp. and *Phytophthora* spp. (116–118). The activity against these soil-borne pathogens comes from the content of antagonistic microorganisms within the AD digestate. For example, *Bacillus* spp. promotes the forming of rhizobacteria due to biotic stress-tolerant spores. Also, *Trichoderma* fungus in AD digestate is reported to suppress a variety of plant pathogenic fungi, such as *Sclerotinia sclerotiorum*, *Pythium ultimum* and *Botrytis cinerea* by colonizing the plant roots and releasing enzymes that degrade fungal cell walls of their competitors (119, 120). This protective activity of the anaerobically produced digestate stands in contrast to the aerobically generated compost, which needs to be sterilized prior to use as the microorganisms contained therein may pose a danger to plants and animals (see section 2).

## 4 Biofuels

Besides the rather crude methods of aerobic or anaerobic digestion of such “mixed” food waste, one may also envisage a more refined collection and thus more directed processing of certain food (waste) items, such as sugars and fats. Indeed, expired or used cooking oils have a long tradition of being used as fuels, with some diesel-powered busses already in the 1980s smelling distinctively of French Fries or Fish & Chips on the go. Today, the large-scale production of “biofuels” such as biodiesel from plant-based oils and bioethanol from sugars is rather common and increasing steadily. Then again, given that agriculture has the potential to provide us with renewable materials year after year, it is not surprising that energy crops, rather than food waste, are used as primary source of biofuels, which is highly controversial from an ecological perspective. Indeed, such “energy plants” are increasingly cultivated in fields where they replace crops traditionally used for animal and human consumption (37). Various examples of energy plants exist, including sugarcane, sugar beets, sunflowers, soybeans, and corn (32).

**Figure 3:**
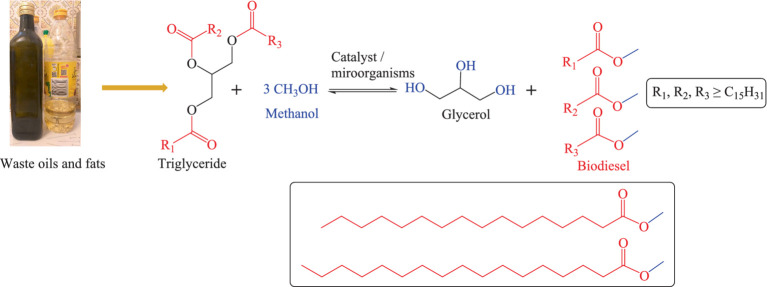
Production of biodiesel via transesterification.

As for biodiesel production, fats and oils, such as algae oil, cooking waste oil, and plant-based oils (among them soybean oil, palm oil, sunflower oil, peanut oil, Jatropha and rapeseed oil) can be hydrolysed and re-esterified through a process known as “transesterification.” As part of the transesterification process, the oils or fats react with an alcohol (mostly methanol) in the presence of a suitable catalyst to form esters and glycerol (121, 122). This process is also exemplified in Figure 3 (123, 124). Essentially, transesterification entails the (bio-)chemical hydrolysis of triglycerides into free fatty acids (FFA) and glycerol, followed by separation of glycerol and esterification of the FFAs with methanol to yield liquid fatty acid esters with high energy density, low melting (−35°C) and high boiling (192°C) points. These esters may serve as biodiesel. The glycerol obtained as a side product in this process is not wasted either – as a “natural product” it finds applications in moisturizers, soaps, cosmetics, and medicines. Microorganisms involved in this biodiesel production process are highly methanol resistant or genetically modified to sustain the harsh chemical conditions and include bacteria such as *Stenotrophomonas maltophilia D18, Lysinibacillus fusiformis B23, Acinetobacter junii C69, Acinetobacter pitti C95, Rhodococcus opacus pd630*, *Pseudomonas citronellolis*, and *Escherichia coli*, as well as yeast species such as *Naganishia liquefaciens* and *Rhodosporidium toruloides* (125–127). It is important to mention that not all types of waste oil need transesterification before being used as a replacement for diesel fuel. Vegetable oils and cooking oils from restaurants or households need transesterification to be converted into biodiesel. The insect oils which are produced through insect-based bioconversion, in contrast, can be used as a replacement for diesel directly, yet with challenges related to cold weather and emissions of pollutants. In general, the total production costs for a gallon of biodiesel can vary from country to country, for instance in 2014 it ranged from 5.53 USD to 6.38 USD in Texas, United States (128). As of April 2023, the price of biodiesel in Germany was 1,471 USD per ton (129). Besides production costs, one should also consider the competition for feedstock resources. This competition, if not managed properly can lead to several adverse consequences (130, 131). The exploitation of edible oils for the production of biodiesel can trigger the “food vs. fuel” debate, diverting valuable agricultural resources from food production to biofuel production which may potentially impact the food prices (132, 133). Moreover, the production of biodiesel is a resource-intensive process which demands excessive amounts of water, energy, and agricultural inputs such as fertilizers and pesticides (130, 131). Biodiesel production offers several advantages, such as reduced greenhouse gas emissions and renewable energy sources, yet one should also consider the competition for feedstock resources and the related challenges to understand the overall situation (130, 131).

Another example of biofuel which can be produced from food waste is bioethanol, in which sugars, especially single sugars, play a vital role. This process typically involves fermentation, relying on microorganisms such as yeasts (*Saccharomyces cerevisiae* and *Issatchenkia orientalis*), bacteria (*Thermoanaerobacter mathranii, Zymomonas mobilis*, and *Geobacillus thermoglucosidasius*), fungi (*Zygomycetes* and *Mucor indicus*), and even algae, such as *Chlorella vulgaris* (37, 134–137). In practice, bioethanol production from food waste involves four main steps. Firstly, the food waste needs to be collected, sorted and pretreated by heating, grinding or shredding to break down complex carbohydrates (136). Secondly, hydrolysis starts to break down complex carbohydrates, mostly cellulose or starch, into simpler sugars, such as xylose or glucose. Thirdly, the fermentation of hydrolysed sugars into ethanol and CO_2_ takes place by microorganisms in the absence of dioxygen as part of an anaerobic fermentation. After fermentation, the ethanol is separated from the fermentation broth via distillation (134).

**Figure 4:**
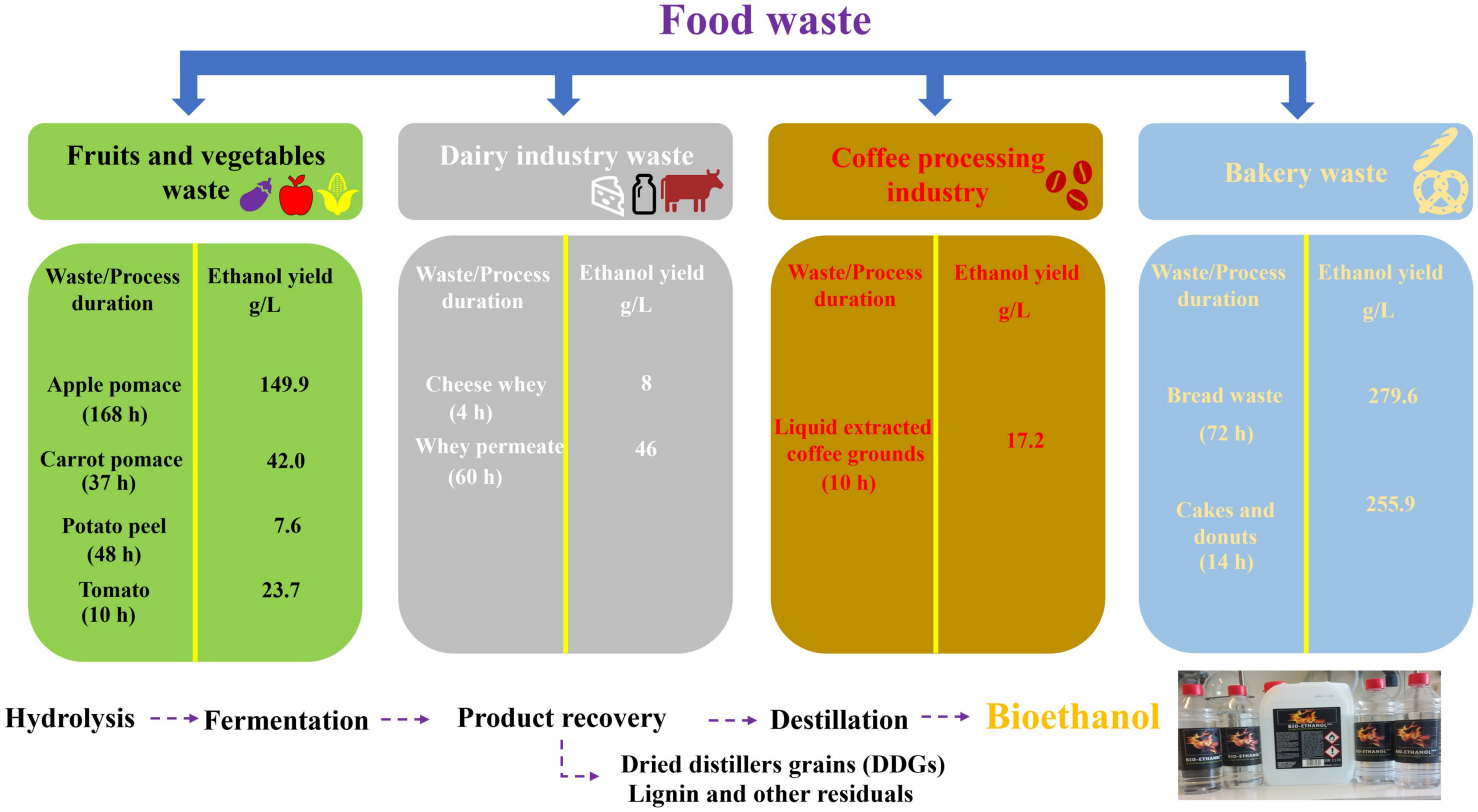
Production of bioethanol from different waste products varies not only in the time required for fermentation but also the yield obtained.

The duration and yield of such a bioethanol production process depend on the type of food waste and microorganisms involved. Vegetable and fruit waste, such as apple pomace, potato and tomato has been reported to yield 149.9 g/L, 7.6 g/L and 23.7 g/L of ethanol, respectively, when fermented in the presence of *Saccharomyces cerevisiae*. Dairy products such as cheese whey yield 8 g/L ethanol when digested by *Kluyveromyces marxianus* (138). The individual bioethanol production phases and more details about the bioethanol yields from different food waste samples are summarized in Figure 4 (138).

In Germany, bioethanol in its pure form is not suitable as petrol-substitute and can only be added to conventional oil-based gasoline at concentrations up to 5% (E5) or 10% (E10) and quite recently even 20% (E20) (139, 140). Brazil has been a pioneer in the use of bioethanol as a fuel source. The “Proalcool” Program, which started in the 1970s, was a key factor in the country’s success in transitioning from fossil fuel to bioethanol (141–143).

Nonetheless, there is also a rather heated ecological debate regarding such renewable “power plants”. On the one side, these plants need to be cultivated and therefore take up valuable agricultural space which is no longer available for the plantation of other plants, for instance wheat (144). In fact, the production of such energy plants in ecologically sensitive regions, for instance in the rainforests, adds to these arguments and issues which need to be resolved first and before such biofuels from cultivars rather than waste can be considered as truly sustainable (144). In addition, the process of bioethanol production is itself energy intensive, especially the distillation step, and encompasses the formation of CO_2_. Indeed, neither biodiesel nor bioethanol, if used in a combustion engine, can be considered as entirely “clean” as burning them in an engine still produces environmental pollutants such as sooth and nitric oxides (NO_x_). Although bioethanol may be used in fuel cells, this technology is still in its infancy. At the same time, bioethanol production poses food security risks, especially in low and middle-income countries. It is essential to strike a balance between utilizing food waste and addressing the global population’s food needs (145, 146). The costs of bioethanol production depend on the type of biomass used, but in general, they range from 0.20 to 0.30 USD per liter (134). Recently, genetic engineering strategies have been employed for the enhancement of biofuel production. Biodiesels, bioalcohols and isoprenoid-based biofuels are, nowadays, obtained by exploiting different genetically engineered yeasts, such as *S. cerevisiae XUSE*, *S. cerevisiae SXA-R2P-E, S. cervisiae M1: kurdriavzevii NUPHS33, S. cerevisiae YG5C423*, *Yarrowia lipolytica, Cryptococcus curvatus* and *Rhodotorula toruloides* (147–152).

## 5 Waste into value: upcycling of industrial food waste (wine, beer, bagasse and beet pulp)

Admittedly, some of the recent examples have been less appetizing, especially for the ones of us who are not yet quite “litterate” or dedicated litter rats yet. They may also have raised the question if initially unwanted biological side products necessarily have to end up in the litter bin or have to be used for applications which are less valuable than the ones associated with the products they are derived from (107, 108). Indeed, in order to fuel the circle of bioeconomy from a truly economical perspective, one may wish to derive at applications for waste - or products from waste - which would be as or even more valuable than the ones of the material itself. In fact, such an increase in value should provide the financial impetus needed for companies to engage in such bio-economic circles (153, 154). Here, re-validation, such as upcycling and bio-valorization, come into play. As the neighbors tends to say: “Are you eventually removing the weed around your house?” – “Not quite, I am collecting my smoke”. At closer inspection, and besides weed(s), a surprising spectrum of organic by-products from food production may be valorized, such as grape seeds, brewed coffee waste, tomato stems, berries, fruits, potatoes, walnut shells and peels (155, 156).

The first and perhaps the most prominent example of industrial food waste is grape pomace which comprises of seeds, stalks and skin and is a high-quality residue of the wine industry laced with flavonoids, anthocyanins and (poly-)phenols (157, 158). The grape pomace contains a higher amount of dietary fibers (19–38%), sugars (12–33%), proteins (8.49–10.32%) and lower amounts of pectin (3.68–29.20%), catechin (150.16 mg) and anthocyanin (84.40-131 mg) per 100 g of pomace based on dry weight (20, 155, 159–161).

**Figure 5:**
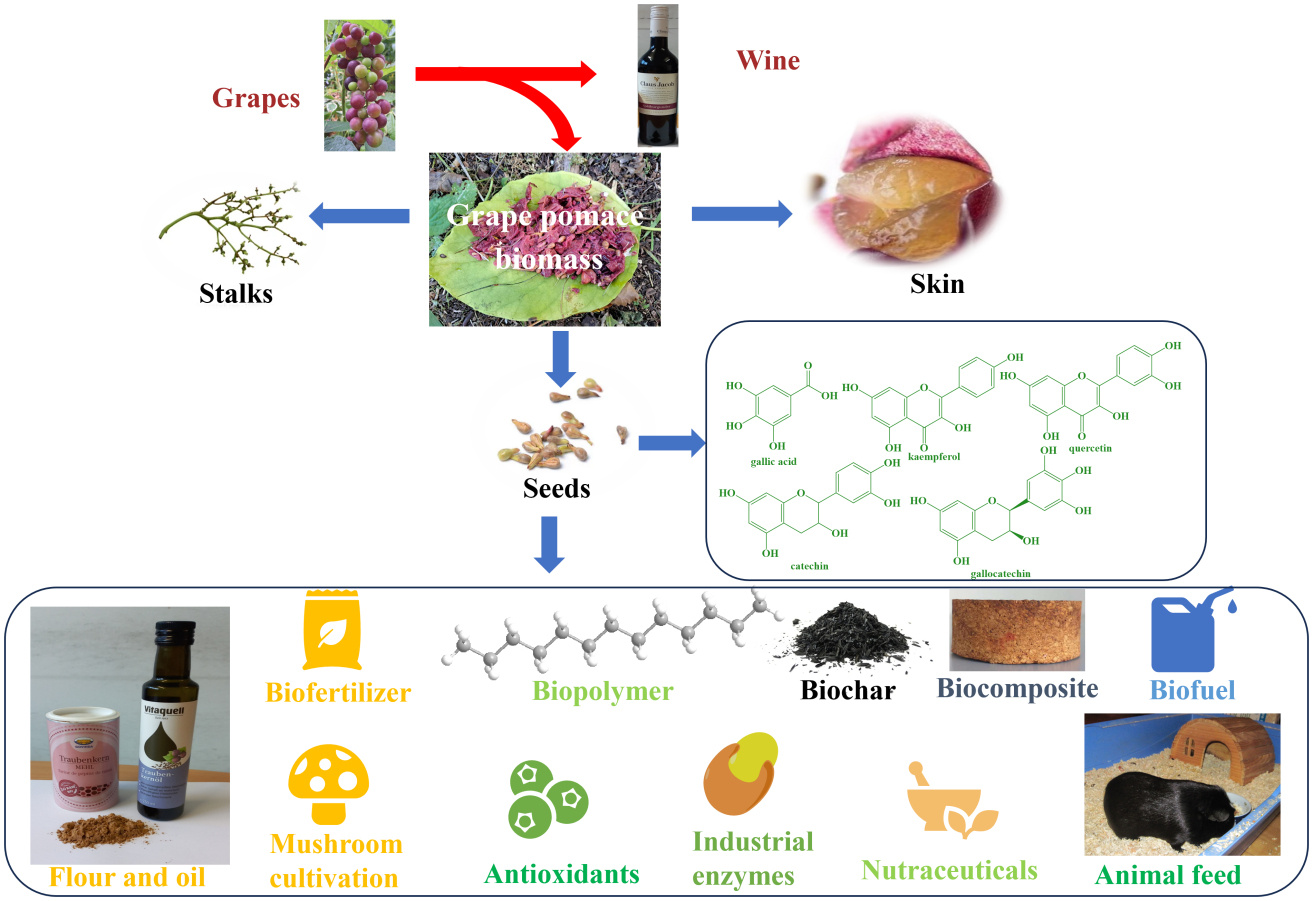
Upcycling of grape seeds for the production of value-added products.

The whole grape pomace can be used or up-cycled to produce energy and various products, such as ruminant feed, biofertilizer, biopolymers, composites, and even a basis for mushroom cultivation through microbial processing, as summarized in Figure 5 (20, 162).

Grape seeds constitute a major part of the grape pomace and for many centuries have been discarded as part of low-value fertilizer (20, 162). Today, the seeds are collected, cleaned and pressed to produce a highly valuable grape seed oil. The increased interest toward grape seeds is primarily due to their high content of phytochemicals, such as phytosterols, carotenoids, vitamin E, unsaturated fatty acids, phenolic compounds (hydroxybenzoic and cinnamic acid derivatives, gallic acid, kaempferol, quercetin, catechin, epicatechin, gallocatechin and procyanidin dimers) (163, 164). Moreover, this oil is rich in *beta*-sitosterol, which has the potential to reduce cholesterol levels and possibly even benign prostatic hyperplasia (BPH, resulting in so-called ewer urinating in men) (165, 166). As for economics, the oil is sold at prices considerably higher than sunflower oil and perhaps even fetches more than the wines the grapes had been processed for initially (20, 161, 163). These days a bottle of reasonable wine in Germany goes for 4 €/L whereas a bottle of grapeseed oil easily catches 8 €/L. (167, 168)

Interestingly, the oil cake from de-oiled grape seeds is no waste either – it may be processed further to grape seed flour as depicted in Figure 5. Again, this flour by itself is a highly valuable product, not only economically, also nutritionally (163, 169, 170). Moreover, the antioxidant and anti-inflammatory properties of de-oiled grape seeds and grape seed flour make them a popular ingredient in cosmetics and personal care products (171). They are often used in the production of anti-aging and skin brightening products (171, 172). Intriguingly, de-oiled grapeseed meal, which is the by-product obtained after extraction of oil, is often also used as a source of protein and dietary fiber in animal feed (171). Besides improving the nutritional value of various food products, the de-oiled grape seeds are often added as a natural preservative to dairy products, processed meats and oils (173–176), thanks to the presence of astringent polyphenolic substances. As for the economics of grape seed flour, 1 kg goes for a staggering 20 € these days.

**Figure 6:**
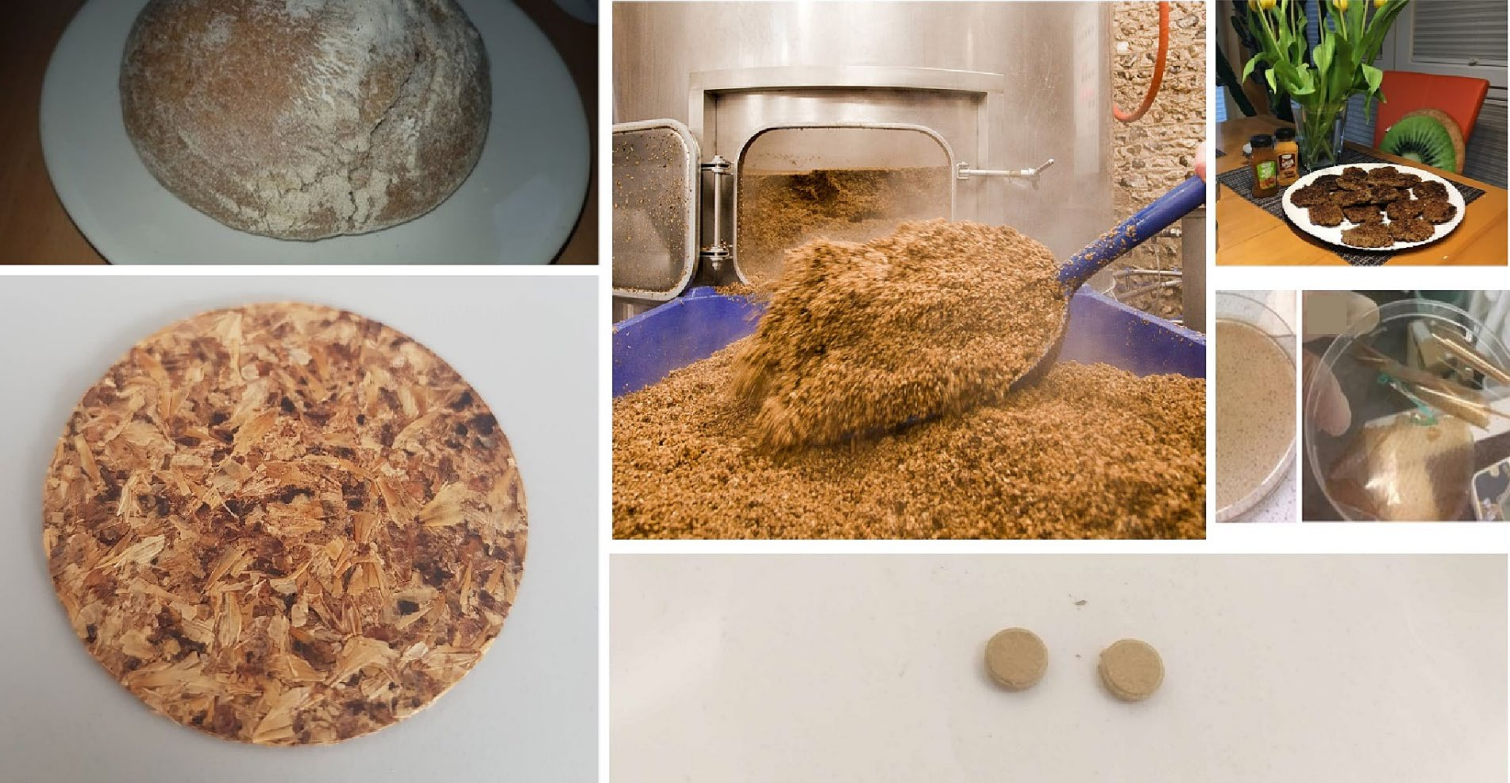
Brewer’s spent grain (BSG, by Homburger Brauhaus, photo generated by the authors) can be employed to produce bread, vegetarian patty or even bioplastics. It can also be pressed to produce sheets or tablets thanks to the presence of lignin which serves as a natural binder.

Brewers spent grain (BSG) is another by-product of the food / beverage industry formed during the process of brewing. The production of 100 L of beer generates 20 kg of BSG which primarily comprises of lignocellulose fibers (around 70% dry weight basis) but is also rich in nutrients such as proteins (20% on a dry weight basis), minerals and vitamins (177). Today 70% of BSG is exploited as feed for animal such as cattle, chicken and poultry, 10% for the production of biogas, and the rest goes to landfills (178) (Figure 6).

Considering these low-value applications, if any, BSG represents another fine example of value gone to waste (179). Indeed, BSG or what is called now occasionally SuperGrain+ (SG+) contains considerable amounts of nutritionally valuable components, such as proteins, fibers and phenolic compounds, which in turn may benefit in some health conditions, such as heart diseases, hypercholesterolemia and cancer (180). Today, it is attractive to process BSG further to produce BSG flour and use it in baking and cooking, in vegan patties and in bread, as a powerful source of dietary fiber and provider of proteins and polyphenols, and as a regulator of the gut microbiota (181).

As for applications outside nutrition, and besides being used as a low-value fuel in biogas plants, BSG can also be processed further to various novel materials, such as solid plates and even bioplastics. BSG plates, for instance, are formed as a result of high temperature and pressure treatment and due to the “melting” and resolidifying of the lignin “glue” (12–28 % w/w) (179). These plates are not only 100% “bio” yet also 100% biodegradable (181). The formation of polymers / plastics, on the other hand, is considerably more complicated and involves acid treatment, similar to the one used to form bioplastics from banana peels (182, 183).

Once again, valorized BSG may be or become more valuable economically and also socially and ecologically than the beer produced in the first instance. And although this is indeed a matter of taste, it shows that such strategies of “turning waste into value” by re-validation may provide the necessary incentives for closing the relevant processing circles in an economically viable fashion turn out to be attractive for local companies, including bakeries, mills and breweries (181). As mentioned before, there is no waste, only material at the wrong place, wrong time and in the wrong hands.

Another example of the valorization of industrial waste are sugar beets which are frequently used to extract sugar, especially in colder climates such as Russia, Germany and the United States, producing 41.2, 31.9 and 8.1 million tonnes of sugar beets per year, respectively (184–186). The production of sugar from beets results in by-products known as Sugar Beet Pulp (SBP). Sugar Beet Pulp Pellets (SBPP), derived from these bulbs, serve as popular livestock feed, suitable for animals such as horses, cattle and sheep (187, 188). The dried form of SBPP, known as “shreds” can be stored for several years without expiring. SBPP is a valuable source of nutrients due to its carbohydrate, fiber and protein content (187, 188). The main carbohydrates present in SBP are sucrose (10% of dry matter) and polysaccharides, including cellulose (22–40%), galactan, araban (24–32%), and pectin (24–32%) (187, 188).

Besides the beverage industry which, not surprisingly, produces millions of tons of by-products every year, there are also other examples of - more limited - food waste turned into value. In fact, some of these materials feed the field of natural cosmetics. Spent coffee grounds (SCG), for instance, is rich in chlorogenic acid and polyhydroxy-alkanoates and apart from being used traditionally in home gardening and against slugs has recently found its way into cosmetics. In this context, it is used as a source of antioxidants or for anti-cellulite activity (189, 190). SCG has also turned into an ingredient of specific hair shampoos as it is still rich in caffeine and thus may be useful in the treatment of androgenetic alopecia. There are even certain bakery products, such as biscuits, which contain SCG (190, 191). Indeed, as for pomace and BSG, SCG has plenty of potential to be used in various ways other than just for slugs in home gardening (192).

The same considerations also apply to the peels of many fruits, especially citrus fruits, such as oranges and lemons. Their peels are increasingly being used in household products, such as cleaning liquids (193). Here, the etherical oils and flavors contained in these peels are of particular importance as they endow these products with natural cleansing properties and fragrances. Notably, these peels also represent a valuable raw material in the field of cosmetics and, if cultivated free of pesticides, can even be processed further by the food industry itself, for instance for the production of jams and marmalades. Again, some of these applications, especially in the field of nutrition, provide such peels with a value which may even exceed the one of the fruits and juices derived from them, such as the famous coarse cut English Orange Marmalade (193, 194). Indeed, one does not have to be a Peelite to admire the use of such peels in exquisite marmalades, cakes, teas and, of course, in cosmetics – and the zero-waste approach that goes with it. Alternatively, the citrus essential oils (CEOs) derived from such peels contain substances such as terpenoids and phenols and thus offer broad-spectrum insecticidal, antifungal and antibacterial properties (195).

Similar opportunities arise for nutshells, which can be processed to abrasive materials, or for ordinary corncobs, which after removing the grains still have a value once dried and milled, for instance as adsorbents (196). Even stale bread can be considered as a valuable raw material and turned into drinks such as Kvass via fermentation by lactic acid bacteria (LAB) (197, 198). Such bacteria can also offer a pickling condition necessary for the manufacture of pickles, green olives, sauerkraut, sausages, buttermilk, yogurt, and some cheeses. Important members include *Streptococcus*, *Lactobacillus*, and *Pediococcus* (197, 199). Another example, LAB such as *Lactiplantibacillus plantarum* and *Lactobacillus acidophilus* bacteria can be used to prepare the fermented Agri-waste for animal feed, often referred to as “silage” (200). Corn silage production requires the waste of corn, i.e., stalks, leaves and the remaining parts of the cobs and LAB such as, *Lactiplantibacillus plantarum*, *Lactobacillus buchneri* and *Weissella hellenica* which may produce a high-quality animal feed able to improve animal physical performance (200, 201).

Eventually, even eggshells may be converted into value-added products. Due to their high content of calcium carbonate (CaCO_3_), eggshells in the form of “eggshell powder” are utilized as calcium supplements for humans. Additionally, their potassium and magnesium content makes them suitable as plant fertilizers (202). Moreover, eggshells can serve as animal feed supplements for livestock. More recently, eggshells have also been employed in natural filters designed to remove impurities and contaminants from waste water (77, 202, 203).

## 6 Bioconversion vs. traditional disposal methods

The previous sections have shown that there are many avenues available to deal with litter, from simple composting in the backyard to sophisticated valorization in shampoos. Notably, the ecological as well as economic impact of these different methods may differ significantly depending on the circumstances. It may be more reasonable, for instance, to simply compost the odd SCG in your garden if you live in a remote village, whereas a highly frequented coffee shop in the center of Paris would probably prefer to donate or even sell the same “waste” to the cosmetics industry – unless, of course, there is a bakery nearby.

In any case, the enormous amounts of food waste produced globally should not simply end up in landfills or incinerators, as they still often do. On the one hand, disposing of food waste in landfills or through incineration has detrimental consequences for the environment (153–155). It leads to greenhouse gas emissions, especially CO_2_ and CH_4_, increased demand for landfill capacity, which affects ecosystems and nearby populations, and contamination of ground- and surface water. The ash generated from incinerating waste and the leachate from landfilling are documented to contain toxic residues eventually worsening air and water pollution and resulting in loss of biodiversity. In contrast, utilizing bioconversion as an alternative method may reduce the negative environmental consequences and extract the value of otherwise hazardous pollutants (202). The food waste management methods, benefits and their impacts on the environment are summarized in Table 3.

**Table 3:** Food waste management methods, their benefits and effects on environment.

Food waste management methods	Gasses produced during the process	Benefits	Impact on environment	References
Composting	CO_2_ Low amounts of CH_4_ and N_2_O	Waste reduction Nutrient soil fertilizer and amendment	Uncontrolled emissions of methane from poorly managed composting Emission of foul odors	(45, 204)
Anaerobic digestion	High amounts of CH_4_ Low amounts of CO_2_ CO, H_2_S, NH_3_ and CH_3_SH	Production of BiogasReduced relianace on fossil fuel Waste management	Emission of methane Delicate balance between energy used and enegry gained	(86, 205, 206)
Biofuels production	CH_3_OH CO_2_	Reduced reliance on fossil fuel Sustainable waste management	Affects air quality High operational costs	(207, 208)
Incineration	High amounts of CO_2_ CO NO_2_ NO SO_2_	Waste reduction Elimination of harmful pathogens and microorganisms	The gaseous emissions can contribute to air pollution and impact local air quality Formation of dioxins from incineration	(209–211)
Disposal in landfills	CH_4_ CO_2_	Waste reduction	Air pollution and climate change Release of odor affects the air quality	(212, 213)

Various strategies should therefore be implemented to minimize food waste, such as food sharing and donations, “lick your plate with pride” campaigns and maximizing recycling and upcycling of food waste wherever and whenever possible. By adopting these alternative approaches, individually or in concert, the environmental impact of food waste disposed in landfills or incinerated may be minimized, thereby contributing to more sustainable waste management practices (202, 214, 215).

## 7 Future trends in waste management

An enormous amount of food, i.e., around 2.5 billion tons, which accounts for almost one third of the food produced annually, goes to the bin. The economic value of this wasted food amounts to approximately 230 billion USD, according to a report by the Boston Consulting Group (BCG) (216). At the same time, one-third of the world’s population is facing the challenge of insufficient food, particularly in developing countries, impacting around 320 million people according to the United Nations. Food waste levels are expected to increase by another third by 2030 (216). In order to counteract the global impacts of food waste, sustainable food waste management approaches have to be adopted. First and foremost important strategies would require waste reduction at the place of origin not only through improved management but also via bio-valorization. Additionally, implementation of advanced recycling and upcycling methods which transform food waste into valuable products, such as bioplastics, fertilizers, and biofuels are equally important (217).

**Figure 7:**
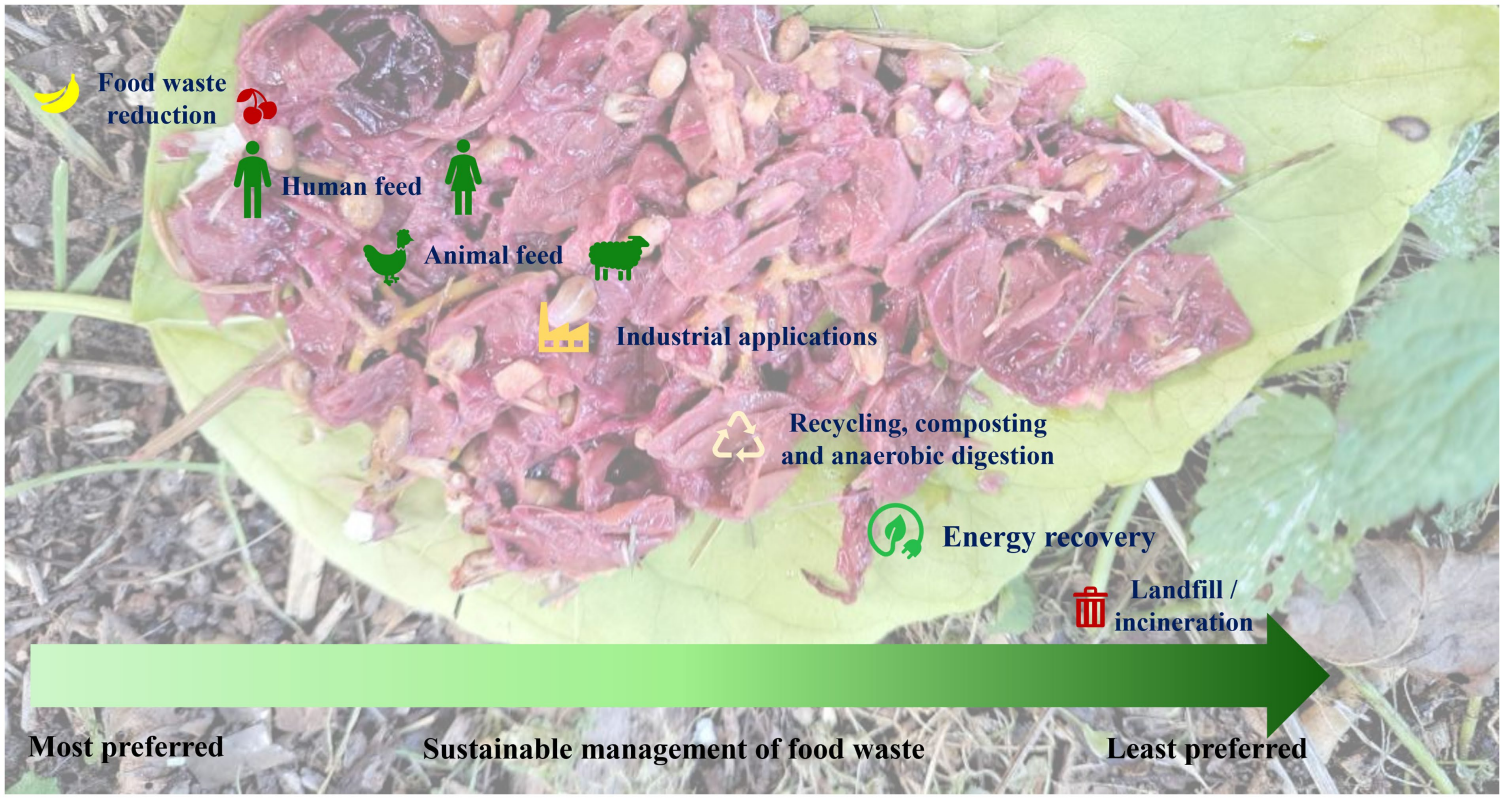
The Food Recovery Hierarchy enlists the actions which can be carried out by the organizations, individuals, and families for the sustainable management of food waste. The highest priority has been assigned to feed humans and animals, followed by industrial applications, recycling, AD and energy recovery. Landfill and/or incineration have been assigned as the least preferred actions.

Sustainable practices, such as composting, anaerobic digestion and insect-based bioconversion of food waste-to-energy are slowly yet gradually evolving and need to be integrated into waste management. Composting is a traditional method, which is becoming more popular not only due to its simplicity but also its ability to produce nutrient-rich soil (44, 45). In contrast, anaerobic digestion is gaining attraction for its capacity to generate biogas for energy production and decreasing waste (101). Insect biorefinery is also another very important emerging tool in the context of the circular bioeconomy since this refinery is able to transform organic waste material to value-added products such as biofertilizers, animal feeds, edible foods, biopolymers, bio-enzymes and biodiesel (76, 218). Furthermore, recent developments in the field of enzyme technology are focused at breaking down the food waste at the molecular level to obtain valuable products (219, 220). Another innovative approach involves utilizing food waste as culture medium to grow algae which provides a sustainable source of biomass for various applications (221, 222). A few strategies involve a combination of techniques for the management or upcycling of organic waste products. Palm bunches have been exploited as biodiesel feedstocks through integrated solid-state and submerged fermentations by fungal co-cultures (223). This technique employs a combination of solid-state and submerged fermentations, with multiple types of fungi working together (fungal co-cultures) for the production of biodiesel (223). Moreover, the extraction of valuable nutrients from organic waste employing supercritical fluids is also an important technique although it may not be cost effective (224). In summary, the future of food waste management is likely to involve a combination of these methods, which will be contingent on local regulations and specific waste streams. The primary focus will revolve around adopting a comprehensive approach that incorporates technological innovation, raises consumer awareness and corporate social responsibility, promotes consumer behavior change and gives more value to surplus “saved” food (225, 226). The Food Recovery Hierarchy outlines actions which organizations, individuals and families can take to prevent and manage wasted food, as summarized in the Figure 7.

## 8 Conclusion

As part of this manuscript, we have aimed to showcase a selection of modern chemical, biochemical and (micro-)biological techniques which may eventually enable us to turn organic by-products or “waste” into valuable (raw) materials and energy. Indeed, valorization of biological (waste) materials currently appears as one of the most promising avenues to limit waste on the one hand and to avoid depletion of natural resources on the other. As one important pillar of a future circular bioeconomy, the “waste into value” concept promises carbon neutrality and also local employment. Rather surprisingly, the underlying strategies of bio-valorization are often based on traditional, robust methods and techniques and therefore may have disappointed the ones expecting high-tech molecular or microbiology. Still, the projects presented are not trivial and often do require modern (bio-)chemistry and analysis to enable a safe, large-scale handling, production, use and, in some cases, consumption.

In future, microbiology, together with molecular biology is likely to improve the fermentation processes mentioned here considerably, for instance in the fields of biofuels and biogas. It may become possible to produce H_2_ rather than CH_4_ via (an)aerobic digestion, thus departing further from carbon-based materials and fuels. Then again, some of these biofuels, such as CH_4_ and bioethanol and most recently ammonia (NH_3_), are easier to store and handle compared to electricity or H_2_, and thus may eventually become amenable to uses in low-emission fuel cells rather than combustion engines.

At the same time, more selective collection and processing methods are likely to increase the yields and thus also value of bio-derived products, such as “green” urea and phosphate from human and animal excrements. Here, methods to collect and refine the waste produced by farm animals more efficiently could be a first step, as it may be easier to implement logistically than returning to the good old family bog hole in the garden.

No doubt, even if driving a honey wagon around town may not be your dream job of the future, new innovations in the field of household waste, from peels to coffee, may indeed provide fascinating opportunities. Citrus peels can already be processed on a small scale and with the help of vinegar to cleaning liquids at home, and decentralized local fermenters and production facilities are springing up and into action even in the smallest villages, such as in Kirkel-Altstadt, Saarland, Germany, where the foundation stone for a new biogas plant running on horse manure and hay has just been laid, and which may soon supply heat and electricity - and odor - for about 300 households (227).

In most cases, a biochemist or biologist is at hand to supervise and control such faculties and to ensure that, literally, no shit happens, from the anecdotal detonation of bog holes to a large-scale escape of BSF. Maybe this simply underlines the fact that numerous challenges for modern biochemistry await us outside the Noble-Prize winning fields of biotechnology and medicine, and despite their low-key, down-to-earth and hands-on applications, these research and development projects are certainly not litter and definitely not for the bin.

## Author contributions

AR: Data curation, Investigation, Formal analysis, Writing – review & editing. ET: Data curation, Formal analysis, Writing – review & editing. AG: Data curation, Formal analysis, Writing – review & editing. AA: Data curation, Formal analysis, Writing – review & editing. MN: Conceptualization, Data curation, Formal analysis, Supervision, Validation, Writing – review & editing. CJ: Conceptualization, Supervision, Validation, Writing – original draft, Writing – review & editing.
